# Supplementary material for: Potential of isoquinoline-alkaloid-rich Papaveraceae extracts against bacteria causing ocular infections – In vitro and In vivo study
Source: Front Pharmacol. 2026 Aug 11;17:1786083. doi: 10.3389/fphar.2026.1786083 (PMC13503538; doi:10.3389/fphar.2026.1786083)
Supplement: Supplementary file 1 [file Supplementaryfile1.docx]

Supplementary

Table 1. Plant material used in a study. BGMP UMW-Botanical Garden of Medicinal Plants, Wroclaw Medical University.

| No. | Species | Part | Plant material origin | GPS | Harvest date | Voucher specimen |
| --- | --- | --- | --- | --- | --- | --- |
| 1 | *Chelidonium majus* | Root | BGMP UMW | 51.11737N  17.07483E | 28.09.2023 | *Ch.m*._R_1–10/2023 |
| 2 | *Chelidonium majus* | Herb | Wrocław , Low Meadow Park | 51.09968N  17.06058E | 09.04.2019 | *Ch.m*._H_1–10/2019 |
| 3 | *Corydalis cheilanthifolia* | Root | Wilamowa | 50.47449N  17.07382E | 11.04.2021 | *C.ch*._R_1–10/2021 |
| 4 | *Corydalis cheilanthifolia* | Herb | Wilamowa | 50.47449N  17.07382E | 11.04.2021 | *C.ch*._H_1–10/2021 |
| 5 | *Corydalis cheilanthifolia* | Herb | BGMP UMW | 51.11737N  17.07483E | 14.04.2022 | *C.ch*._H_1–10/2022 |
| 6 | *Glaucium flavum* | Root | BGMP UMW | 51.11737N  17.07483E | 28.09.2023 | *G.f.*_R_1-3/2023 |
| 7 | *Glaucium flavum* | Herb | BGMP UMW | 51.11737N  17.07483E | 19.06.2023 | *G.f.*_H_1-3/2023 |
| 8 | *Fumaria officinalis* | Herb | Wrocław , Low Meadow Park | 51.09968N  17.06058E | 16.03.2023 | *F.o*._H_1-3/2023 |

Reference standards used in a study:

1. Chelidonina (Extrasynthese, France, cat. No. 0603; CAS 476-32-4)
2. Sanguinarine (Extrasynthese, France, cat. No. 1845; CAS 5578-73-4)
3. Allocryptopine (Merck, Germany; cat. No. SMB01042; CAS 24240-04-8)
4. Berberine (Extrasynthese, France, cat. No. 0601; CAS 633-65-8)
5. Coptisine (PhytoLab, Germany; cat No. 89524; CAS 6020-18-4)
6. Chelerythrine (Extrasynthese, France, cat. No. 1559; CAS 3895-92-9)
7. Protopine (Extrasynthese, France, cat. No. 1586; CAS 130-86-9)
8. Tetrahydopalmatine, PhytoLab, France, cat No. 89807, CAS 2934-97-6
9. Palmatine, Merck, Germany, cat. No. 361615, CAS 171869-95-7
10. Glaucine, TRC, cat No. TRC-G406902, CAS 475-81-0

Figure 1. HPLC-DAD chromatogram of alkaloids (acquired at 280 nm) in extracts of *C. majus* root.

berberine

coptisine

chelidonine

allocryptopine

protopine

Figure 2. HPLC-DAD chromatogram of alkaloids (acquired at 280 nm) in extracts of *C. majus* herb.

chelidonine

Figure 3. HPLC-DAD chromatogram of alkaloids (acquired at 280 nm) in extracts of *C. cheilanthifolia* root.

coptisine

protopine

protopine derivative

Figure 4. HPLC-DAD chromatogram of alkaloids (acquired at 280 nm) in extracts of *C. cheilanthifolia* herb.

coptisine

berberine

protopine derivative

protopine

Figure 5. HPLC-DAD chromatogram of alkaloids (acquired at 280 nm) in extracts of *C. cheilanthifolia* herb.

berberine

protopine

protopine derivative

Figure 6. HPLC-DAD chromatogram of alkaloids (acquired at 280 nm) in extracts of *G. flavum* root.

tetrahydropalmatine

chelidonine

glaucine derivative 1

protopine

Figure 6. HPLC-DAD chromatogram of alkaloids (acquired at 280 nm) in extracts of *G. flavum* herb.

glaucine drivative 1

glaucine drivative 2

glaucine

Figure 7. HPLC-DAD chromatogram of alkaloids (acquired at 280 nm) in extracts of *F.officinalis* herb.

protopine

Table 2. The data used for identification of the investigated compounds.

| Compound | Retention Time | Formula | Structure | UV-Vis Spectrum |
| --- | --- | --- | --- | --- |
| Protopine | 10.881 | C_20_H_19_NO_5_ |  |  |
| Allo-cryptopine | 12.300 | C_21_H_23_NO_5_ |  |  |
| Glaucine | 12.611 | C_21_H_25_NO_4_ |  |  |
| Chelidoni-ne | 12.774 | C_21_H_23_NO_5_ |  |  |
| Tetrahydropalmatine | 13.29 | C_21_H_25_NO_4_ |  |  |
| Coptisine | 13.766 | C_19_H_14_NO_4_^+^ |  |  |
| Sanguinari-ne | 19.028 | C_20_H_14_NO_4_ |  |  |
| Palmatine | 19.312 | C_21_H_21_NO_4_ |  |  |
| Berberine | 19.880 | C_19_H_14_NO_4_^+^ |  |  |
| Chelerythri-ne | 26.775 | C_21_H_18_NO_4_ |  |  |

| No. | Reference substance | Calibration curve equation | R^2^ |
| --- | --- | --- | --- |
| 1 | Protopine | y=6.409+6.097 | 0.98700 |
| 2 | Allocryptopine | y=7.911x+13.1799 | 0.98433 |
| 3 | Glaucine | y=11.520-18.703 | 0.99440 |
| 4 | Chelidonine | y=10.744+29.904 | 0.98411 |
| 5 | Tetrahydropalmatine | y=8.684+4.291 | 0.99815 |
| 6 | Coptisine | y=8.742x+10.961 | 0.99317 |
| 7 | Sanguinarine | y=13.376-31.142 | 0.98555 |
| 8 | Palmatine | y=20.943x-55.249 | 0.98995 |
| 9 | Berberine | y=15.536-6.272 | 0.99194 |
| 10 | Chelerythrine | y=32.291-10.284 | 0.99464 |
